# Characterizing self-assembled structures made with magnetic Janus nanoparticles

**DOI:** 10.1107/S2052252524001532

**Published:** 2024-02-29

**Authors:** Elizabeth Blackburn

**Affiliations:** a Division of Synchrotron Radiation Research, Department of Physics, Lund University, SE-22100 Lund, Sweden

**Keywords:** nanoscience, small-angle X-ray scattering, nanostructure, magnetic Janus particles, magnetic field induced orientations, X-ray photon correlation spectroscopy, anisotropic scattering, particle dynamics

## Abstract

Small-angle X-ray scattering has revealed how magnetic Janus particles pair up in solutions in small and large magnetic fields.

The cells of our bodies maintain their integrity through their cell membranes. These membranes are made up of molecules with a cap that is hydro­philic (attracted to water) and a tail that is hydro­phobic (repelled by water). These molecules self-assemble when in water to form a sheet with the hydro­phobic tails pointing inwards, creating a region with almost no water. This process creates the leak-tight membranes that protect our cells.

Casagrande *et al.* (1989 ▸) were able to make spherical nanoparticles where one side was hydro­philic and the other hydro­phobic. The difference between the two sides was immediately visible when exposed to water, with one-half of the particle wetting completely, and the other being covered by small droplets of water. These particles can be trapped at the interface between oil and water, and, in his Nobel lecture, de Gennes (1991 ▸) speculated that these particles might lead to boundaries that, unlike cell membranes, do permit for some exchange between the two sides, through the gaps between the particles, so that like the two-faced Roman god Janus, they stand watch over doorways.

From this initial work, there has been a great effort to develop bespoke Janus particles, where the two sides can differ in many ways. By coating one side with a metal, the application of an alternating electric field causes them to move. If the metal is ferromagnetic, like nickel, then this motion can be directed using a magnetic field, and the controlled motion of these Janus particles has been demonstrated, and the particles used to transport and deliver small cargoes like polystyrene particles (Demirörs *et al.*, 2018 ▸) and potentially bacteria or viruses (Demirörs *et al.*, 2017 ▸).

Now, a recent publication in **IUCrJ** by Manna *et al.* (2024 ▸) has validated a structural model of the magnetic Janus particles that will be broadly applicable to all particles of this type. This work was driven initially by the observation of highly anisotropic scattering patterns in ultra-small-angle X-ray scattering experiments on silica particles with part of the surface coated with ferromagnetic nickel, in particular a pattern dubbed ‘butterfly like’ at higher magnetic fields (*e.g.* 1 T), when the magnetic field is applied perpendicular to the X-ray beam (Zinn *et al.*, 2023 ▸).

At small magnetic fields, dynamical studies by Zinn *et al.* (2023 ▸) using X-ray photon correlation spectroscopy on the small-angle scattering show that the particles display Brownian motion, even as the individual particles align with the magnetic field. As the field is increased, the anisotropy of the scattering increases significantly, and this was originally interpreted (Zinn *et al.*, 2023 ▸) as the alignment of all magnetic caps with the field direction, and chain-like assembly. However, this model could not capture the anisotropy correctly, and so Manna *et al.* developed a mathematical model for the individual magnetic Janus particles, from which the X-ray scattering profile could be calculated. With this basis in place, they found that the only way to properly describe the observed patterns as the magnetic field was increased was by positing the formation of a pair, or dimer, of two Janus particles, with their magnetic caps facing each other.

At higher fields, these Janus pairs cluster together to form chain-like arrangements, although with significantly more potential complexity possible, as these pairs are expected to form intertwined chains from packing considerations. This result will have consequences for considerations of using magnetic Janus particles as cargo shuttles, as the applied magnetic field level will have to be carefully considered, as well as the consequences for this type of arrangement in a varying electric field. This work also indicates that very low fields can affect the nature of the interactions quite significantly, which is of importance in ensuring robust environments for repeatable controlled behaviour.

The X-ray scattering results do not interact directly with the magnetic properties of the particle, instead detecting the structural anomaly that is the magnetic cap. This means that the actual magnetic alignment associated with these particular nanoparticles is not known. Manna *et al.*’s work finds that the particles orient so that the symmetry axis of the particle with cap lies parallel to the field, but with the nickel caps in contact in the formation of the Janus pairs. There is an implicit assumption that the magnetic moment associated with the caps is parallel to the magnetic field in both cases – this follows from considering the interaction between two magnetic dipoles in a magnetic field.

In other magnetic Janus particles, a variety of structures and orientations have been identified, and the methods outlined by Manna and co-workers provide an avenue for testing how common these structures are over a large sample in suspension. With these methods, for example, it should be possible to track the evolution of the staggered chain structures seen in iron-capped spheres by Smoukov *et al.* (2009 ▸), by appropriate adjustment of the Janus pair model to account for the stepped capping. It may even be possible by using small-angle neutron scattering to probe the magnetic signal directly to confirm the proposed magnetic moment directions given in Manna *et al.* (2024 ▸) and Smoukov *et al.* (2009 ▸).
